# Outer Nuclear Layer as the Main Predictor to Anatomic Response to Half Dose Photodynamic Therapy in Chronic Central Serous Retinopathy

**DOI:** 10.1155/2019/5859063

**Published:** 2019-10-13

**Authors:** Keissy Sousa, Ana Rita Viana, Joana Pires, Carla Ferreira, Lara Queirós, Manuel Falcão

**Affiliations:** ^1^Ophthalmology Department, Hospital de Braga, Braga, Portugal; ^2^Ophthalmology Department, Centro Hospitalar Universitário S. João, Porto, Portugal; ^3^Medicine School, Universidade do Minho, Braga, Portugal; ^4^Ophthalmology Department, Hospital Cuf Porto, Porto, Portugal; ^5^Department of Surgery and Physiology, Faculty of Medicine of the University of Porto, Porto, Portugal

## Abstract

**Purpose:**

To evaluate the predictors for subretinal fluid resorption in patients with chronic central serous retinopathy (cCSR) submitted to half-dose photodynamic therapy (HD-PDT).

**Methods:**

Observational, longitudinal, and retrospective study of patients with cCSR submitted to HD-PDT in a tertiary ophthalmology department in Portugal between January 2015 and February 2018. Best-corrected visual acuity (BCVA) and SD-OCT at baseline and 12 ± 3 months after treatment were performed. The central macular thickness (CMT), outer nuclear layer (ONL) thickness, integrity of the external limiting membrane (ELM), ellipsoid (EZ) and interdigitation zone (IZ), subretinal fluid (SFR) height, and choroidal thickness (CT) were evaluated. Patients were classified into responders and nonresponders based on SRF resorption.

**Results:**

Sixty-one eyes of 42 patients were included; 75.4% were classified as responders. Final BCVA was significantly better in responders (*p*=0.002). The baseline ONL was thicker (*p* < 0.01) and intact ELM (67.2% vs. 16.4%), EZ (49.2% vs. 8.2%), and IZ (31.2% vs. 1.6%) were more prevalent in responders than in nonresponders. Anatomic response was correlated with a thicker ONL (rs (59) = 0.416, *p*=0.001^*∗*^), intact ELM (rs (59) = 0.261, *p*=0.04^*∗*^), EZ (rs (59) = 0.278, *p*=0.03^*∗*^), and IZ (rs (59) = 0.318, *p*=0.01^*∗*^). Binary logistic regression showed that a thicker ONL thickness increased the chance of an anatomic response to HD-PDT. The other evaluated retinal layers did not have statistical significance in the binary regression model.

**Conclusions:**

cCSR responders to HD-PDT have a better final BCVA, a thicker baseline ONL, and an intact baseline ELM, EZ, and IZ. However, ONL was the only predictor in a logistic regression model for SRF resorption.

## 1. Introduction

Central serous retinopathy (CSR) is characterized by macular serous detachment in the absence of other ocular abnormalities [1, 2]. Although most acute cases resolve spontaneously, the recurrence or persistence of serous subretinal fluid can lead to deterioration in vision [2, 3]. One definition of chronic CSR (cCSR) is characterized by persistent serous retinal detachment for more than 6 months [4]. Over the last decades, fluorescein angiography (FA) and indocyanine green angiography (ICG) have been used to confirm the diagnosis. However, these techniques remain insufficient for predicting visual outcomes [3]. The recent advances in spectral-domain optical coherence tomography (SD-OCT) have made it possible to detect changes in the retinal microstructure [2, 3]. The ability to evaluate changes in central macular thickness, submacular fluid, thickness of the outer nuclear layer (ONL), and thickness of the external retinal bands such as the external limiting membrane (ELM), the ellipsoid (EZ), and interdigitation (IZ) zones has increased our knowledge on the pathophysiology of CSR [2–10]. Subfoveal choroidal thickness (CT) has been reported to be thickened in CSR likely due to choroidal vascular dilation [11]. SD-OCT evaluation of the subretinal fluid has become an important tool for the management of the disease as it provides fast and innocuous characterization of disease activity, determining which patients require treatment due to persistent subretinal fluid, and it allows the anatomical evaluation of treatment response.

In cases of persistent subretinal fluid, half-dose photodynamic therapy (HD-PDT) with verteporfin has been widely used to treat CSR by inducing choroidal vascular remodeling and decreasing choroidal vascular permeability with a high anatomical success [4, 8, 12, 13]. However, there has been a discrepancy between anatomic success of the treatment, usually defined as absence of subretinal fluid, and visual recovery. This fact suggests that there may be prognostic factors that can predict anatomic and visual recovery [4, 12, 14]. The aim of our study is to identify baseline SD-OCT characteristics that may predict the anatomic improvement after HD-PDT in patients with chronic CSR. We evaluated the CMT, ONL, ELM, EZ, IZ, subretinal fluid (SRF), and CT as prognostic factors for subretinal fluid resorption about 12 months after HD-PDT treatment.

## 2. Methods

Medical records of patients who had the diagnosis of cCSR that were submitted to HD-PDT between January 2015 and February 2018 were reviewed for this retrospective study. All investigations were performed in accordance with the principles of the Declaration of Helsinky and approved by the Ethics Committee of Hospital de Braga. All patients with a diagnosis of cCSR who performed HD-PDT during the reported period were included. Data were collected at baseline and 12 ± 2.6 months after treatment. No other treatments for cCSR including mineralocorticoid receptor antagonists or focal laser were performed during the study period. Patients with myopia ≥6.0 diopters and macular disorders such as choroidal neovascularization, polypoidal choroidal vasculopathy, age-related macular degeneration, history of vitreomacular disease, intravitreal anti-VEGF (vascular endothelial growth-factor) or mineralocorticoid-receptor antagonists treatment, laser photocoagulation or prior PDT ≤ 6 months, cataract, or optical media opacity that restricted the examination of ocular fundus were excluded from this study.

Each study participant underwent a comprehensive ophthalmologic examination, SD-OCT, and FA (TRC-50DX, Topcon Medical Systems, Inc., Tokyo, Japan) and/or ICG (TRC-50DX, Topcon Medical Systems, Inc., Tokyo, Japan) evaluation. Demographic data collected included sex, age, time of diagnosis, laterality, and previous treatments. Ophthalmologic examination included best-corrected visual acuity (BCVA) and slit-lamp biomicroscopy. BCVA was assessed using the decimal scale chart and converted to logarithm of the minimum angle of resolution (log MAR).

Spectralis SD-OCT (Heidelberg Engineering, Heidelberg, Germany) was used to measure retinal thickness and evaluate the outer retinal layers. The OCT imaging technique consisted in obtaining a macular square (20 × 20°) composed of 25 horizontal B-scans, spaced at 240 *μ*m. Each B-scan was averaged 9 times (ART 9). Additionally, for each case, a single horizontal and a single vertical B-scan using the enhanced depth imaging mode, averaged 100 times (ART 100), and centered on the fovea was obtained.

The SD-OCTs were performed immediately before treatment and 12 ± 3 months later. The presumed foveal center was determined as the area lacking the inner retinal layers in the macular region. Data collected were: central macular thickness (CMT) and the following foveal parameters: outer nuclear layer (ONL) thickness, presence or absence of external limiting membrane (ELM), ellipsoid band (EZ), interdigitation band (IZ), subretinal fluid height (SRF), and choroidal thickness (CT). ONL was defined as the distance between the inner limiting membrane and the ELM at the central fovea. SRF was measured as the hyporeflective space from the IZ to the RPE. Choroidal thickness was measured subfoveally with enhanced depth imaging, from the outer portion of the retinal pigment epithelium (RPE) to the inner surface of the sclera. When there was any disruption of the central foveal millimeter of the ELM, EZ, and IZ bands, they were classified as absent; when these layers were preserved, they were classified as present. All measurements and evaluations were made by two independent investigators (K.S. and A.R.V.) on the horizontal high quality scans centered on the fovea. Any prominent difference between the two investigators was discussed with the senior author (M.F.), and the reconciled measurement was recorded.

PDT with verteporfin (Visudyne®, Novartis, Basel, Switzerland) was performed using a half-dose protocol. Verteporfin at 3 mg/m^2^ dose was infused for 8 min; diode laser light (689 nm) was delivered (Visulas 690 D, Carl Zeiss Meditec Inc., Jena, Germany) for 83 s. The standard dose of laser intensity (600 mW/cm^2^) and fluence was used (50 J/cm^2^). The spot size was determined by the area size of the maximum leakage point on ICG or FA.

Two different groups of patients were created for the analysis. Patients were divided into anatomic responders (R) and nonresponders (NR) based on SRF resorption. Patients were classified as responders if the SRF improved equal or greater than 50% resorption of SRF measured on SD-OCT height.

All statistical analysis was performed using SPSS software version 25.0 (SPSS Inc., Chicago, IL), and *p* values <0.05 were considered statistically significant. All values were presented as mean (±standard deviation (SD)) or median (interquartile range (IQR)). Nonparametric tests were applied after nonnormality of the sample was confirmed by the Shapiro–Wilk test. Wilcoxon signed-rank test was used to compare baseline and posttreatment visual acuity. Spearman's rho correlation was used to find the associations between anatomic response and baseline OCT characteristics. Mann–Whitney *U* or independent sample *t*-test was used to compare differences between groups. To identify factors affecting treatment success, we performed a binary univariate analysis and subsequently a multivariate regression using the backward conditional method to exclude possible confounders and analyzed the baseline variables to verify any predictive factors.

## 3. Results

Seventy eyes of 47 patients were selected. Nine eyes were excluded due to lack of complete follow-up. Sixty-one eyes of 42 patients were included. Mean age was 55.7 ± 12.6 years; 71.4% (*n* = 30) were male. Median time from diagnosis until treatment was 20 (IQR 26) months. Corticoid use was present in 27.9% (*n* = 11) of patients. Naïve eyes were 44.3% (*n* = 27), and previous treatments, such as HD-PDT, mineralocorticoids, or anti-VEGF, were applied in 55.7% (*n* = 34) cases outside the study period and at least 3 months before the study.  Table 1 shows demographic characteristics of our sample.

### 3.1. Anatomic Response and Visual Acuity

The samples were divided in two groups: R and NR, regarding the SRF improvement on SD-OCT. Forty-six patients (75.4%) were classified as R and 15 (24.6%) were classified as NR. Overall, the median baseline BCVA was 0.30 logMAR (IQR 0.40), and the final BCVA was 0.20 logMAR (IQR 0.30), which meant a significant visual improvement (*z* = −2.85, *p*=0.004). In the NR group, there was no significant variation in median BCVA throughout the study, from 0.70 (IQR 0.6) to 0.40 (IQR 0.50) (*z* = −0.12, *p*=0.91). In the R group, the median baseline BCVA improved from 0.25 log MAR (IQR 0.20) to 0.20 log MAR (IQR 0.2) (*z* = −3.56, *p* < 0.001). Baseline visual acuity was not statistically different between both groups (*U* = 242, *z* = −1.75, *p*=0.08). However, final BCVA was better in R (0.20 logMAR (IQR 0.20) than NR (0.40 logMAR (IQR 0.50)) (*U* = 156, *z* = −3.08, *p*=0.002).

### 3.2. OCT Parameters

The baseline SD-OCT parameters evaluated in R and NR are shown in Table 2. The baseline ONL was thicker in the R group (43.5 ± 22.9 *μ*m) than in the NR group (23.9 ± 32.2 *μ*m, *p* < 0.01). The ELM integrity was more prevalent in the R group than in the NR group (67.2% vs. 16.4%), *p*=0.04. The same was observed within the EZ (49.2% vs. 8.2%, *p*=0.03) and the IZ (31.2% vs. 1.6%, *p*=0.01).

### 3.3. Independent Correlations with Anatomic Response

The correlation between the baseline retinal layers and the anatomic response to HD-PDT was evaluated as shown in Table 3. The ONL thickness and the presence of the other outer retinal layers are significantly correlated with the final anatomic response (ONL (rs (59) = 0.416, *p*=0.001^*∗*^), ELM (rs (59) = 0.261, *p*=0.04^*∗*^), EZ (rs (59) = 0.278, *p*=0.03^*∗*^), and IZ (rs (59) = 0.318, *p*=0.01^*∗*^)). Overall, neither baseline BCVA and time from symptoms/diagnosis until treatment were correlated with the anatomic effect (rs (63) = 0.08, *p*=0.54).

### 3.4. Binary Regression on Anatomical Response

A binomial multivariate logistic regression was performed to ascertain the effect of final BCVA, ONL, EZ, and IZ on the likelihood that patients will respond to HD-PDT. The logistic regression model was statistically significant (χ^2^(4) = 11.7, *p*=0.002). The model explained 17.4% to 25.9% (Nagelkerke *R*^2^) of the variance of anatomical response and correctly classified 78.7% of cases. Of the four predictive values, only one was statistically significant, ONL (*p*=0.02). The increase of 1 *μ*m of ONL thickness elevates 1.04 times the chance to anatomic response to HD-PDT. All other variables lost statistical significance in the multivariate logistic regression model as shown in Table 4.

## 4. Discussion

Due to the increasing costs of medical therapy, the decision to treat patients in clinical practice has led to an increasing concern over cost-effectiveness. Understanding which patients will benefit from treatment in expensive medications such as PDT is of paramount importance. Evaluating the anatomy of the outer retina before treatment may help predict the anatomic response. In our series, though all external layers had an independent significant correlation with the anatomical fluid resorption, we observed that patients with a thicker ONL had a higher likelihood to obtain an adequate anatomic response to HD-PDT in the dicotomic model. This could mean that ONL status might be the most important layer to predict the anatomic response.

Current advances in the SD-OCT technology have provided precious information regarding the importance of outer retinal layers in visual function in eyes with CSR [6, 14, 15]. Improvement in BCVA and SRF after HD-PDT is well documented [4, 14, 16–19] and was confirmed in this study. We also showed that when an anatomic response does not occur, we do not find a significant change in vision. Treatment effect based on SRF resorption and CMT reduction has been used to classify the response to PDT [18, 20]. Most of the studies reported an anatomical success of 60–85%, which was in line with our results of 75.4% of anatomical improvement [14, 18, 19]. In our study, we disclose that the anatomic responders had a final better median BCVA (with less improvement) as in the study of Matuskova et al. [4]. This could also be an indicator that using the PDT when the retina is not yet dysfunctional may lead to better SFR resorption, CMT improvement, and improved visual acuity. However, we did not find that time to treatment was an indicator of anatomic improvement after treatment. Chung et al. and Iacono et al. reported similar findings as they described that the duration of symptoms was not linked with subretinal fluid resolution [21, 22]. This could mean that retinal dysfunction in this disease may not be linked to the amount of time in which subretinal fluid is present and could be linked to other outer retinal changes that occur but are not time-dependent. It is possible that the biochemical characteristics of subretinal fluid may differ from patient to patient, and these differences may have different effects on the external retinal layers that have been described [2, 12, 21].

In our study, the R group had different baseline SD-OCTs from NR. They had a thicker ONL, and the ELM, EZ, and IZ were more frequently intact. However, in our binary regression model, we found that the ONL thickness was the only predictor of SRF resorption. The ONL is the innermost retinal layer of the variables in our study. The ONL is composed by the nuclei of photoreceptors. Thinning of this layer could be a marker of definite retinal damage that may predict a poor response to PDT. When the subretinal fluid is present, the RPE is not able to absorb the tip of the outer segment. This may lead to both an elongation of photoreceptor outer segments and possibly photoreceptor cell apoptosis with subsequent thinning of ONL [9, 23, 24]. Other studies have reported that the ONL thickness could be an important predictor of visual acuity after one year of half-fluence PDT [8, 25]. However, these studies differ from the present studies because they had a lower number of eyes (22 and 36) and they used half-fluence PDT instead of half-dose PDT.

Other authors have reported other results but, in their studies, the ONL was not assessed, and therefore the importance of this layer could not be evaluated. A strong correlation between the disruption of the ellipsoid (previously denominated IS/OS) and low BCVA and a decrease in visual acuity when the ELM is disrupted was found in untreated chronic CSR [3]. One other study did not find any predictive factors with final BCVA, including ELM or the ellipsoid zone when low-fluence PDT was used [4]. When conventional PDT was used, a disruption of the EZ, longer duration of visual symptoms, and RPE atrophy negatively impacted on visual function [14]. Finally, Chung et al. used SRF resolution as a prognostic factor for visual acuity improvement for HD-PDT. They also observed that EZ was not a prognostic factor for SFR resorption, but they did not include other variables than the EZ as predictors [21]. These data suggest that evaluating the EZ as the sole predictive factor for visual function for chronic CSR might be reductionist and that all outer retinal structures, especially the ONL, should be taken into account as a predictive factor and in future evaluations of response to therapy.

Our study also has limitations. It is a retrospective study. There was an asymmetry between the number of patients in the two groups because the majority of patients had a good response. There were a significant number of patients who were followed-up for a long time, and other treatments have been performed before, which could explain the poorer results. Mean age was higher than usual, and it could be linked for the longer time of disease and due to 31.9% of patients that had cCSR linked to corticoid use. Patients were followed up in a regular clinical setting without strict follow-up protocols and treatment indications as clinical trials; however, it tries to reflect everyday clinical practice.

This is the first study to analyze the anatomic characteristics of all the outer retinal layers in a multivariable model to try and identify predictors of SRF resorption in patients treated with HD-PDT for cCSR with one year of follow-up. Anatomic recovery is usually proportional to visual acuity improvement [10, 14, 21]. That was the main reason to study predictors that may influence anatomic recovery. Evaluation of the outer retinal layers especially the ONL may help predict which patients may have an anatomical response to HD-PDT. Treating patients with a thin ONL may not have a good anatomical response and therefore will not obtain significant visual benefits.

## 5. Conclusion

HD-PDT is one treatment option for cCSR. Multiple studies used assorted variables trying to predict a better result of PDT in cCSR [25]. Most studies have evaluated single retinal layers using SD-OCT. We analyzed all the outer retinal layers, and in our model, a thicker ONL was the best predictor for better anatomic results.

## Figures and Tables

**Table 1:** Demographic characteristics and clinical data.

Variable	(42 patients, *n* = 61)	
Sex (male/female)	30/12	
Age (years)	55.7 ± 12.6	
Time until treatment (months)	20 (IQR 26)	
Previous treatments	HD-PDT	16.4%
	Mineralocorticoid	1.6%
	MPL	3.3%
	Anti-VEGF	4.9%
	Multiple	29.5%

values are presented as mean ± standard deviation in age and as median (IQR = interquartile range) in time until treatment.

**Table 2:** Variables at baseline from responders and nonresponders groups.

Variables	Responders	Nonresponders	
Best-corrected visual acuity (log MAR)	0.25	0.7	*U* = 242, *z* = −1.75, *p*=0.08
Central macular thickness (*μ*m)	315.5 (183)	283 (95)	*U* = 417, *z* = 1.06, *p*=0.29
Outer nuclear layer (*μ*m)	43.5 ± 22.9	23.9 ± 32.2	*t*(59) = −2.59, *p*=0.01^*∗*^
External limiting membrane (present/absent)	41/5	10/5	*p*=0.04^*∗*^
Ellipsoid zone (present/absent)	30/16 (65.2/34.8%)	5/10 (33.3%/66.7%)	*p*=0.03^*∗*^
Interdigitation zone (present/absent)	19/27 (41.3%/58.7%)	1/14 (6.7/93.3%)	*p*=0.01^*∗*^
Choroidal thickness (*μ*m)	304.2 ± 74.1	282.3 ± 47.8	*t*(59) = −1.08, *p*=0.29
Subretinal fluid (*μ*m)	96.5 (201)	61 (89)	*U* = 384, *z* = 0.66, *p*=0.51

Variables at baseline included in the analysis from responders and nonresponders groups. There was a significant difference between both groups in outer nuclear layer, external limiting membrane, ellipsoid zone, and interdigitation zone. ^*∗*^Statistically significant.

**Table 3:** Independent correlations between variables at baseline and anatomic response.

Independent variables at baseline	Correlation with anatomic response
Best-corrected visual acuity	rs (59) = −0.23, *p*=0.08
Time from diagnosis	rs (57) = 0.05, *p*=0.73
Central macular thickness	rs (59) = 0.15, *p*=0.25
Outer nuclear layer	rs (59) = 0.416, *p*=0.001^*∗*^
External limiting membrane	rs (59) = 0.261, *p*=0.04^*∗*^
Ellipsoid zone	rs (59) = 0.278, *p*=0.03^*∗*^
Interdigitation zone	rs (59) = 0.318, *p*=0.01^*∗*^
Choroidal thickness	rs (59) = −0.098, *p*=0.45
Subretinal fluid	rs (59) = 0.085, *p*=0.52

Independent correlations between the anatomic response and the baseline retinal layers or baseline best-corrected visual acuity (BCVA). There is an independent correlation between baseline outer nuclear layer, external limiting membrane, ellipsoid zone, and interdigitation zone. rs = Spearman's rho correlation; *p* = *p* value; ^*∗*^ = statistically significant.

**Table 4:** Univariate and multivariate binomial regression to estimate the probability of anatomic response.

Variable	Univariate analysis Exp(B)	Univariate analysis *p*-value, 95% CI	Multivariate analysis, Exp(B)	Multivariate analysis *p*-value, 95% CI
Baseline best-corrected visual acuity	0.151	*p*=0.04, 0.025 − 0.891^*∗*^	0.49	*p*=0.49, 0.065 – 3.66
Time until diagnosis	1.005	*p*=0.73, 0.976 – 1.035	X	
Central macular thickness	1.001	*p*=0.76, 0.997 – 1.005	X	
Outer nuclear layer	1.04	*p*=0.02, 1.01 – 1.07^*∗*^	1.04	*p*=0.02, 1.007 – 1.071^*∗*^
External limiting membrane	0.244	*p*=0.05, 0.059 – 1.008	X	
Ellipsoid zone	0.267	*p*=0.04, 0.078 − 0.915^*∗*^	1.27	*p*=0.76, 0.268 – 6.029
Interdigitation zone	0.102	*p*=0.03, 0.012 − 0.839^*∗*^	0.16	*p*=0.1, 0.018 – 1.43
Choroidal thickness	1.005	*p*=0.28, 0.996 – 1.014	X	
Subretinal fluid	1.001	*p*=0.71, 0.996 – 1.005	X	

To estimate the probability of anatomic response of baseline variables, a univariate analysis of each variable to exclude possible confounders was performed initially, and subsequent multivariate analysis was performed. Only the outer nuclear layer was a significant predictor. *∗*, statistically significant; X, not included.

## Data Availability

The data used to support the findings of this study are restricted by the Ethics Committee of Hospital de Braga in order to protect patient privacy. Data are available for researchers who meet the criteria for access to confidential data.
